# Diabetes predisposition inherited through maternal germline

**DOI:** 10.1002/ctm2.969

**Published:** 2022-07-08

**Authors:** Hong Zhu, Yi Cheng, Hefeng Huang

**Affiliations:** ^1^ Obstetrics and Gynecology Hospital, Institute of Reproduction and Development Fudan University Shanghai China; ^2^ Research Units of Embryo Original Diseases Chinese Academy of Medical Sciences Shanghai China; ^3^ Key Laboratory of Reproductive Genetics (Ministry of Education) Zhejiang University School of Medicine Hangzhou China; ^4^ Shanghai Key Laboratory of Embryo Original Diseases Shanghai China

1

The sustained global rise in the prevalence of diabetes mellitus over the last decades increasingly affects young adults. Thus, 7%–20% of pregnancies are complicated by maternal diabetes.^1^ ‘Diabetic mothers give birth to diabetic babies’ is one oversimplified conclusion from human and animal studies demonstrating that maternal hyperglycaemia predisposes the offspring to the development of diabetes.^2^ However, little is known about the molecular mechanisms underlying this phenomenon known as ‘metabolic memory’. A recent article published in *Nature* reveals that glucose intolerance – a sign of prediabetic status – is transmitted from a diabetic mother to the next generation via the female oocytes.

Mounting evidence has shown that maternal diabetes during the gestational period can make offspring more likely to increase their risk of diabetes.^3^ To distinguish the effect of pre‐gestation and lactation, the authors fertilized the oocytes from STZ‐induced hyperglycaemia female mice in vitro with healthy male sperm and transferred the 2‐cell embryo to normal glycaemia foster female mice. The approach of in vitro fertilization and embryo transfer enables the researchers only to focus on the effect of oocytes on metabolic memory transmission.

The mice generated from hyperglycaemic oocytes had normal growth trajectories but exhibited impaired glucose tolerance compared with controls. The authors showed that glucose intolerance in offspring was attributed to impaired glucose‐stimulated insulin secretion by pancreatic islets beta cells. This glucose intolerance was more pronounced in male than female offspring, which worsened with age and high‐fat diet in both sexes, suggesting that F1 generation inherited the diabetes susceptibility from the hyperglycaemia‐exposed oocytes. Interestingly, this metabolic phenotype was detected only in F1 offspring, not persisted in F2 generation (at least without metabolic phenotype till 24‐week old), indicating that maternal hyperglycaemia had an intergenerational rather than a transgenerational effect on offspring health via oocytes. Given that when sperm exposed to a prediabetic status, the transgenerational metabolic inheritance was observed in F2 offspring via paternal germline, indicating that the maternal germline‐dependent diabetes inheritance differs from paternal germline.^4^

One general mechanism by which early life exposures could be linked to acquired traits is the alteration of epigenetic marks. In mammals, efficient genome‐wide DNA methylation reprogramming occurs in the pre‐implantation embryo and the germline, and these two rounds of epigenetic erasure are required to remove the acquired epigenetic signatures to ensure that the embryo properly reflects the genetic blueprint characteristic of each species.^5^ Epigenetic reprogramming is two sides of the same coin. Reprogramming events act as natural barriers aimed precisely at maintaining the pluripotency of the zygote and ensuring proper embryo development.^6^ However, each reprogramming window shows active epigenetic regulators and unstable epigenetic markers,^7^ which processes are susceptible to any environmental factors and can be transmitted to the following generation. Thus, one possibility is that maternal hyperglycaemia might disturb the epigenome during post‐fertilization, leading to acquired traits of glucose intolerance in offspring. To test the hypothesis, researchers first compared the mRNA transcriptome of oocytes from the hyperglycaemia mice and controls. Among all the differentially expressed genes, *Tet3* transcripts were detected significantly downregulated in hyperglycaemia oocytes. *Tet3* expression was also decreased in oocytes from another diabetic mouse model and diabetic women.

The mammalian TET family includes three members, TET1, TET2, and TET3, which can catalyse the conversion of 5‐position of cytosine (5mC) to 5‐hydroxymethylcytosine (5hmC).^8^ TET3 is abundantly expressed in mature oocytes and acts as a maternal factor critical for paternal DNA demethylation.^9^ Investigators found that the oxidation of methylated DNA level was markedly lower in the paternal pronuclei of zygotes from hyperglycaemic mice. To evaluate the effects of reduced maternal TET3 and paternal genome 5hmC on offspring glucose metabolism, the authors carried out whole‐genome bisulfite sequencing on offspring E18.5 pancreas. Numerous regions were hypermethylated in foetal pancreas generated from hyperglycaemia oocytes, and differentially hypermethylated regions (hyper‐DMRs) in the promoters of several genes involved in insulin secretion, including a pancreas‐specific promoter that drives pancreatic transcription of *Gck*. *Gck* encodes glucokinase, a key rate‐limiting enzyme that regulates glucose‐stimulated insulin secretion.^10^

The authors went on to show the hyper‐DMRs of *Gck* derived from the paternal genome and persisted in a developmental stage from pre‐implantation embryo to postnatal adulthood. However, it is difficult to exclude the possibility that TET3 is also involved in the maternal demethylation of metabolic genes. To explore whether reduced maternal *Tet3* expression was the only contributor to *Gck* hypermethylation and glucose intolerance in offspring, they generated a *Tet3* knockout mouse, in which *Tet3* expression was insufficient due to oocytes‐specific *Tet3* heterozygous and homozygous knockout, mimicked the effect of maternal hyperglycaemia. Offspring from genetically engineered *Tet3^+/−^* and *Tet3^−/−^* oocytes exhibited altered DNA methylation status of *Gck* and glucose intolerance, which was similar to those from hyperglycaemia oocytes. Additionally, a supplemental injection of exogenous *Tet3* mRNA to mature oocytes abolished the effects of hyperglycaemia or *Tet3* deletion.

Previous results showed that paternal germline was susceptible to metabolic challenges,^4^ and this study provided evidence that female germline was also vulnerable to environmental factors when they are maturing. High glucose exposure led to a significant reduction in Tet3 mRNA levels in mice and human oocytes during in vitro maturation, due to accelerating Tet3 mRNA degeneration. This finding in germline cells coincided with somatic cells exposed to hyperglycaemia,^11^ reflecting that high glucose possibly causes *Tet3* destabilization. It will be exciting to explore the mechanisms by which molecular events control the *Tet3* mRNA or protein stability.

Taken together, this study revealed maternal and paternal co‐contribution to oocytes origin of diabetic susceptibility. Hyperglycaemia directly compromised maternal oocytes’ *Tet3* levels and further indirectly defected paternal DNA methylation reprogramming via TET3 insufficiency, finally causing the perturbations of insulin secretion gene expression and glucose intolerance in next generation (Figure 1). Future research is required to determine whether the TET3‐mediated inheritance is relevant to humans and, if so, the mechanism and molecular targets by which high glucose regulates TET3 stability and expression will hopefully revolutionize preventative and therapeutic approaches for diabetes management. As famously said by the English poet Alexander Pope, ‘The proper study of Mankind is Man’.

**FIGURE 1:**
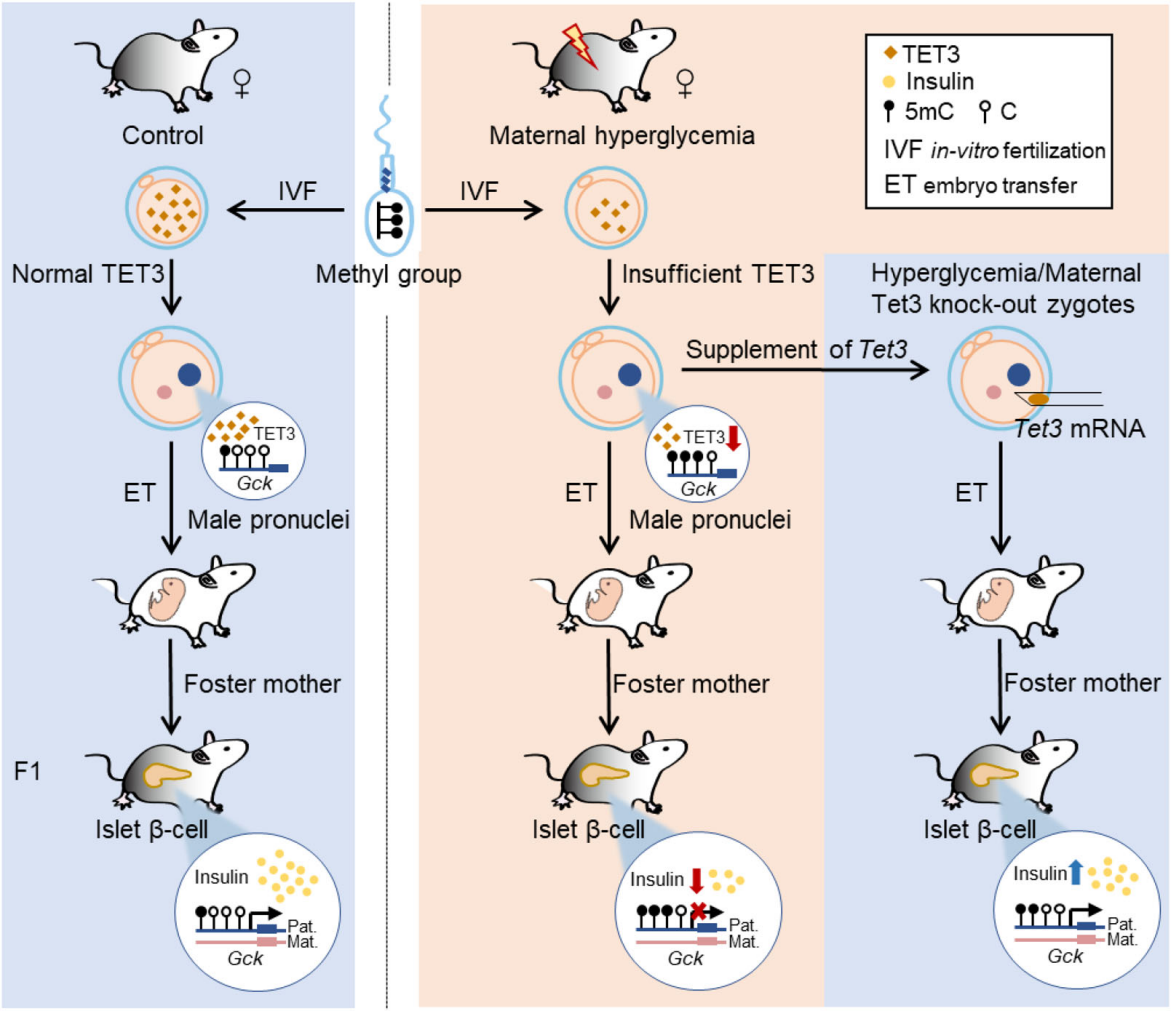
Maternal germline mediates the inheritance of glucose intolerance via TET3. The genome of sperm is tagged with many methyl groups, and paternal DNA occurs to demethylation after fertilization. If the oocytes during maturation exposed to maternal hyperglycaemia, the demethylation enzyme of TET3 decreases, which disrupts paternal genome demethylation in the fertilized oocytes. When the F1 generation grows to adults, the lower transcription of genes such as *Gck* in the pancreas islets leads to defective insulin secretion. The supplement of exogenous *Tet3* mRNA to oocytes with hyperglycaemia exposure or *Tet3* deletion abolished the effects of Tet3 insufficiency and improved insulin secretion in offspring.

## CONFLICT OF INTEREST

The authors declare that there is no conflict of interest that could be perceived as prejudicing the impartiality of the research reported.
